# Supplementary material for: Modeling glioblastoma heterogeneity as a dynamic network of cell states
Source: Mol Syst Biol. 2021 Sep 16;17(9):e10105. doi: 10.15252/msb.202010105 (PMC8444284; doi:10.15252/msb.202010105)
Supplement: Supplementary file 6 — Source Data for Figure 5 [file MSB-17-e10105-s004.zip › Figure5A_sourcedata/GSEA_3017/nosigdb_stateD.GseaPreranked.1622795010397/RICHARDS_NOWAKOWSKI_SCIENCE_2017_MGE_IPC2_UPREG.html]

Details for gene set RICHARDS\_NOWAKOWSKI\_SCIENCE\_2017\_MGE\_IPC2\_UPREG[GSEA]

|  || Dataset | state23017 |
| Phenotype | NoPhenotypeAvailable |
| Upregulated in class | na\_neg |
| GeneSet | RICHARDS\_NOWAKOWSKI\_SCIENCE\_2017\_MGE\_IPC2\_UPREG |
| Enrichment Score (ES) | -0.45693284 |
| Normalized Enrichment Score (NES) | -2.7074971 |
| Nominal p-value | 0.0 |
| FDR q-value | 0.0 |
| FWER p-Value | 0.0 |
Table: GSEA Results Summary

  

Fig 1: Enrichment plot: RICHARDS\_NOWAKOWSKI\_SCIENCE\_2017\_MGE\_IPC2\_UPREG      
 Profile of the Running ES Score & Positions of GeneSet Members on the Rank Ordered List

  

| PROBE | GENE SYMBOL | GENE\_TITLE | RANK IN GENE LIST | RANK METRIC SCORE | RUNNING ES | CORE ENRICHMENT || 1 | PTTG1 |  |  | 14 | 0.490 | -0.0090 | No |
| 2 | DSEL |  |  | 16 | 0.472 | -0.0060 | No |
| 3 | ARL4C |  |  | 24 | 0.422 | -0.0090 | No |
| 4 | HDGF |  |  | 58 | 0.338 | -0.0371 | No |
| 5 | HMGB3 |  |  | 80 | 0.311 | -0.0541 | No |
| 6 | SOX4 |  |  | 91 | 0.298 | -0.0610 | No |
| 7 | TROAP |  |  | 109 | 0.283 | -0.0745 | No |
| 8 | CHD7 |  |  | 116 | 0.278 | -0.0778 | No |
| 9 | BIRC5 |  |  | 130 | 0.271 | -0.0877 | No |
| 10 | NUP205 |  |  | 165 | -0.251 | -0.1174 | No |
| 11 | ZNF519 |  |  | 166 | -0.251 | -0.1153 | No |
| 12 | TICRR |  |  | 199 | -0.253 | -0.1431 | No |
| 13 | SMARCA2 |  |  | 203 | -0.254 | -0.1437 | No |
| 14 | MKI67 |  |  | 206 | -0.254 | -0.1435 | No |
| 15 | RBM23 |  |  | 231 | -0.257 | -0.1638 | No |
| 16 | PIK3R3 |  |  | 277 | -0.261 | -0.2037 | No |
| 17 | RBL1 |  |  | 285 | -0.262 | -0.2080 | No |
| 18 | FUS |  |  | 290 | -0.263 | -0.2096 | No |
| 19 | ZEB2 |  |  | 317 | -0.265 | -0.2317 | No |
| 20 | KNTC1 |  |  | 329 | -0.267 | -0.2397 | No |
| 21 | ARID1A |  |  | 335 | -0.267 | -0.2422 | No |
| 22 | C18orf54 |  |  | 345 | -0.268 | -0.2484 | No |
| 23 | EXOSC8 |  |  | 347 | -0.268 | -0.2471 | No |
| 24 | CTDSPL2 |  |  | 358 | -0.269 | -0.2542 | No |
| 25 | PRR11 |  |  | 361 | -0.269 | -0.2538 | No |
| 26 | RNASEH2B |  |  | 370 | -0.270 | -0.2590 | No |
| 27 | KAT2B |  |  | 373 | -0.271 | -0.2586 | No |
| 28 | USP33 |  |  | 375 | -0.271 | -0.2573 | No |
| 29 | RIF1 |  |  | 403 | -0.274 | -0.2803 | No |
| 30 | TUBB |  |  | 410 | -0.274 | -0.2836 | No |
| 31 | CCDC150 |  |  | 413 | -0.275 | -0.2832 | No |
| 32 | MASTL |  |  | 414 | -0.275 | -0.2809 | No |
| 33 | CEP57 |  |  | 418 | -0.275 | -0.2814 | No |
| 34 | NUP107 |  |  | 422 | -0.276 | -0.2819 | No |
| 35 | UHRF1 |  |  | 438 | -0.278 | -0.2936 | No |
| 36 | TOPBP1 |  |  | 439 | -0.278 | -0.2913 | No |
| 37 | LCORL |  |  | 464 | -0.281 | -0.3114 | No |
| 38 | POLH |  |  | 467 | -0.282 | -0.3109 | No |
| 39 | CEP97 |  |  | 474 | -0.282 | -0.3141 | No |
| 40 | APOLD1 |  |  | 476 | -0.282 | -0.3127 | No |
| 41 | FOXM1 |  |  | 478 | -0.282 | -0.3113 | No |
| 42 | ECT2 |  |  | 481 | -0.283 | -0.3108 | No |
| 43 | DLX1 |  |  | 490 | -0.284 | -0.3159 | No |
| 44 | FBXW7 |  |  | 494 | -0.284 | -0.3164 | No |
| 45 | ZGRF1 |  |  | 499 | -0.285 | -0.3177 | No |
| 46 | HAUS6 |  |  | 510 | -0.286 | -0.3247 | No |
| 47 | MCM10 |  |  | 515 | -0.287 | -0.3260 | No |
| 48 | SESTD1 |  |  | 528 | -0.288 | -0.3349 | No |
| 49 | CDK6 |  |  | 538 | -0.290 | -0.3409 | No |
| 50 | KCTD9 |  |  | 541 | -0.290 | -0.3403 | No |
| 51 | CENPO |  |  | 550 | -0.291 | -0.3454 | No |
| 52 | WDR76 |  |  | 557 | -0.292 | -0.3485 | No |
| 53 | WDHD1 |  |  | 559 | -0.292 | -0.3470 | No |
| 54 | NUP155 |  |  | 566 | -0.293 | -0.3502 | No |
| 55 | PSRC1 |  |  | 574 | -0.295 | -0.3543 | No |
| 56 | RFC4 |  |  | 575 | -0.295 | -0.3518 | No |
| 57 | BTG3 |  |  | 581 | -0.296 | -0.3540 | No |
| 58 | NEDD4L |  |  | 595 | -0.299 | -0.3637 | No |
| 59 | SPAG5 |  |  | 605 | -0.301 | -0.3696 | No |
| 60 | RFC2 |  |  | 607 | -0.302 | -0.3680 | No |
| 61 | NCAPH2 |  |  | 616 | -0.303 | -0.3730 | No |
| 62 | MCM3 |  |  | 625 | -0.305 | -0.3779 | No |
| 63 | CREB1 |  |  | 644 | -0.310 | -0.3921 | No |
| 64 | PRC1 |  |  | 645 | -0.310 | -0.3896 | No |
| 65 | ARHGAP11B |  |  | 650 | -0.310 | -0.3907 | No |
| 66 | CDK5RAP2 |  |  | 651 | -0.311 | -0.3881 | No |
| 67 | DNMT1 |  |  | 659 | -0.312 | -0.3921 | No |
| 68 | RAD21 |  |  | 674 | -0.314 | -0.4025 | No |
| 69 | HAUS8 |  |  | 678 | -0.315 | -0.4027 | No |
| 70 | CHAF1A |  |  | 679 | -0.315 | -0.4001 | No |
| 71 | TMPO |  |  | 688 | -0.317 | -0.4049 | No |
| 72 | PLK4 |  |  | 699 | -0.319 | -0.4116 | No |
| 73 | KIF4A |  |  | 706 | -0.320 | -0.4146 | No |
| 74 | MPHOSPH9 |  |  | 713 | -0.321 | -0.4175 | No |
| 75 | ST8SIA5 |  |  | 737 | -0.327 | -0.4363 | No |
| 76 | TMSB4X |  |  | 742 | -0.329 | -0.4372 | No |
| 77 | SPC24 |  |  | 747 | -0.330 | -0.4382 | No |
| 78 | HADH |  |  | 754 | -0.333 | -0.4411 | No |
| 79 | PRIM2 |  |  | 764 | -0.336 | -0.4467 | No |
| 80 | UBR7 |  |  | 767 | -0.336 | -0.4458 | No |
| 81 | BRD8 |  |  | 778 | -0.338 | -0.4523 | No |
| 82 | MCM4 |  |  | 783 | -0.339 | -0.4532 | No |
| 83 | TMEM245 |  |  | 788 | -0.340 | -0.4541 | Yes |
| 84 | CDC25C |  |  | 792 | -0.341 | -0.4541 | Yes |
| 85 | CDC20 |  |  | 793 | -0.341 | -0.4512 | Yes |
| 86 | ZNF680 |  |  | 796 | -0.342 | -0.4502 | Yes |
| 87 | FANCI |  |  | 801 | -0.345 | -0.4511 | Yes |
| 88 | CEP192 |  |  | 802 | -0.345 | -0.4482 | Yes |
| 89 | AHI1 |  |  | 806 | -0.347 | -0.4481 | Yes |
| 90 | BRCA2 |  |  | 808 | -0.347 | -0.4462 | Yes |
| 91 | HMGXB4 |  |  | 809 | -0.347 | -0.4433 | Yes |
| 92 | NCAPD2 |  |  | 811 | -0.347 | -0.4413 | Yes |
| 93 | BLM |  |  | 812 | -0.348 | -0.4384 | Yes |
| 94 | CENPM |  |  | 818 | -0.349 | -0.4402 | Yes |
| 95 | MAD2L1 |  |  | 819 | -0.350 | -0.4373 | Yes |
| 96 | BUB3 |  |  | 826 | -0.351 | -0.4400 | Yes |
| 97 | PCM1 |  |  | 834 | -0.353 | -0.4436 | Yes |
| 98 | SRBD1 |  |  | 836 | -0.354 | -0.4416 | Yes |
| 99 | CBX5 |  |  | 842 | -0.356 | -0.4433 | Yes |
| 100 | CENPJ |  |  | 849 | -0.357 | -0.4459 | Yes |
| 101 | RFC3 |  |  | 852 | -0.358 | -0.4448 | Yes |
| 102 | RND3 |  |  | 853 | -0.358 | -0.4418 | Yes |
| 103 | ARL6IP6 |  |  | 854 | -0.358 | -0.4388 | Yes |
| 104 | GAS2L3 |  |  | 856 | -0.359 | -0.4368 | Yes |
| 105 | STK39 |  |  | 858 | -0.359 | -0.4347 | Yes |
| 106 | NEK2 |  |  | 861 | -0.359 | -0.4336 | Yes |
| 107 | CCDC15 |  |  | 883 | -0.367 | -0.4502 | Yes |
| 108 | CENPQ |  |  | 884 | -0.367 | -0.4471 | Yes |
| 109 | SMC1A |  |  | 885 | -0.367 | -0.4440 | Yes |
| 110 | STIL |  |  | 888 | -0.369 | -0.4428 | Yes |
| 111 | RAD18 |  |  | 898 | -0.374 | -0.4481 | Yes |
| 112 | CKAP2 |  |  | 900 | -0.375 | -0.4459 | Yes |
| 113 | TK1 |  |  | 902 | -0.375 | -0.4438 | Yes |
| 114 | SAP30 |  |  | 905 | -0.376 | -0.4425 | Yes |
| 115 | DST |  |  | 913 | -0.379 | -0.4459 | Yes |
| 116 | TUBB4B |  |  | 916 | -0.380 | -0.4446 | Yes |
| 117 | DEPDC1B |  |  | 923 | -0.383 | -0.4470 | Yes |
| 118 | DSN1 |  |  | 926 | -0.385 | -0.4457 | Yes |
| 119 | MSH2 |  |  | 927 | -0.385 | -0.4424 | Yes |
| 120 | USP1 |  |  | 930 | -0.386 | -0.4411 | Yes |
| 121 | HAT1 |  |  | 933 | -0.387 | -0.4397 | Yes |
| 122 | PSIP1 |  |  | 934 | -0.388 | -0.4365 | Yes |
| 123 | KIF18B |  |  | 935 | -0.388 | -0.4333 | Yes |
| 124 | DIAPH3 |  |  | 943 | -0.390 | -0.4366 | Yes |
| 125 | CENPC |  |  | 945 | -0.391 | -0.4342 | Yes |
| 126 | SKA1 |  |  | 950 | -0.395 | -0.4347 | Yes |
| 127 | TMEM237 |  |  | 953 | -0.396 | -0.4333 | Yes |
| 128 | CDCA7L |  |  | 954 | -0.397 | -0.4299 | Yes |
| 129 | DTL |  |  | 956 | -0.397 | -0.4276 | Yes |
| 130 | SKA3 |  |  | 965 | -0.399 | -0.4317 | Yes |
| 131 | XPO1 |  |  | 967 | -0.400 | -0.4293 | Yes |
| 132 | HIRIP3 |  |  | 970 | -0.402 | -0.4278 | Yes |
| 133 | RTKN2 |  |  | 973 | -0.403 | -0.4264 | Yes |
| 134 | FANCD2 |  |  | 976 | -0.405 | -0.4249 | Yes |
| 135 | YEATS4 |  |  | 979 | -0.407 | -0.4233 | Yes |
| 136 | FANCL |  |  | 981 | -0.407 | -0.4209 | Yes |
| 137 | DCLK2 |  |  | 986 | -0.408 | -0.4212 | Yes |
| 138 | SMCHD1 |  |  | 990 | -0.411 | -0.4206 | Yes |
| 139 | POLD3 |  |  | 1002 | -0.415 | -0.4274 | Yes |
| 140 | KIF11 |  |  | 1003 | -0.416 | -0.4240 | Yes |
| 141 | CDH2 |  |  | 1007 | -0.418 | -0.4233 | Yes |
| 142 | MIS18A |  |  | 1020 | -0.427 | -0.4309 | Yes |
| 143 | CENPL |  |  | 1021 | -0.427 | -0.4274 | Yes |
| 144 | TIMELESS |  |  | 1024 | -0.429 | -0.4257 | Yes |
| 145 | NCAPG2 |  |  | 1027 | -0.430 | -0.4239 | Yes |
| 146 | POLQ |  |  | 1033 | -0.436 | -0.4250 | Yes |
| 147 | TPX2 |  |  | 1034 | -0.437 | -0.4214 | Yes |
| 148 | LIG1 |  |  | 1035 | -0.437 | -0.4177 | Yes |
| 149 | CEP152 |  |  | 1040 | -0.439 | -0.4178 | Yes |
| 150 | FANCB |  |  | 1044 | -0.443 | -0.4169 | Yes |
| 151 | DDB2 |  |  | 1045 | -0.443 | -0.4132 | Yes |
| 152 | NCAM1 |  |  | 1049 | -0.445 | -0.4123 | Yes |
| 153 | KIF22 |  |  | 1051 | -0.446 | -0.4095 | Yes |
| 154 | BRIP1 |  |  | 1052 | -0.447 | -0.4058 | Yes |
| 155 | ZBTB20 |  |  | 1061 | -0.451 | -0.4095 | Yes |
| 156 | SMC2 |  |  | 1062 | -0.452 | -0.4058 | Yes |
| 157 | MCM8 |  |  | 1064 | -0.452 | -0.4029 | Yes |
| 158 | EZH2 |  |  | 1070 | -0.455 | -0.4038 | Yes |
| 159 | MMS22L |  |  | 1072 | -0.457 | -0.4009 | Yes |
| 160 | DEPDC1 |  |  | 1074 | -0.457 | -0.3981 | Yes |
| 161 | HIST1H3D |  |  | 1076 | -0.458 | -0.3952 | Yes |
| 162 | GRIK3 |  |  | 1078 | -0.459 | -0.3923 | Yes |
| 163 | MND1 |  |  | 1083 | -0.461 | -0.3922 | Yes |
| 164 | POLA1 |  |  | 1088 | -0.464 | -0.3920 | Yes |
| 165 | ATAD5 |  |  | 1091 | -0.465 | -0.3900 | Yes |
| 166 | ITGB3BP |  |  | 1096 | -0.467 | -0.3899 | Yes |
| 167 | ASCL1 |  |  | 1097 | -0.467 | -0.3860 | Yes |
| 168 | ZWINT |  |  | 1099 | -0.467 | -0.3830 | Yes |
| 169 | ARHGEF26 |  |  | 1110 | -0.477 | -0.3884 | Yes |
| 170 | BUB1B |  |  | 1111 | -0.477 | -0.3844 | Yes |
| 171 | RACGAP1 |  |  | 1112 | -0.479 | -0.3804 | Yes |
| 172 | MGME1 |  |  | 1113 | -0.479 | -0.3764 | Yes |
| 173 | E2F8 |  |  | 1116 | -0.485 | -0.3743 | Yes |
| 174 | BARD1 |  |  | 1117 | -0.486 | -0.3702 | Yes |
| 175 | TTK |  |  | 1119 | -0.487 | -0.3671 | Yes |
| 176 | RBBP8 |  |  | 1128 | -0.494 | -0.3705 | Yes |
| 177 | NCAPD3 |  |  | 1130 | -0.496 | -0.3673 | Yes |
| 178 | JADE1 |  |  | 1141 | -0.503 | -0.3724 | Yes |
| 179 | PCNA |  |  | 1142 | -0.504 | -0.3682 | Yes |
| 180 | VRK1 |  |  | 1143 | -0.505 | -0.3640 | Yes |
| 181 | IDH2 |  |  | 1146 | -0.508 | -0.3616 | Yes |
| 182 | CKAP2L |  |  | 1147 | -0.508 | -0.3574 | Yes |
| 183 | TMEM106C |  |  | 1148 | -0.509 | -0.3532 | Yes |
| 184 | FAM111B |  |  | 1151 | -0.514 | -0.3508 | Yes |
| 185 | NUSAP1 |  |  | 1154 | -0.515 | -0.3483 | Yes |
| 186 | GMNN |  |  | 1155 | -0.516 | -0.3440 | Yes |
| 187 | CEP135 |  |  | 1156 | -0.517 | -0.3397 | Yes |
| 188 | DBF4 |  |  | 1157 | -0.520 | -0.3354 | Yes |
| 189 | CCNF |  |  | 1160 | -0.522 | -0.3329 | Yes |
| 190 | MIS18BP1 |  |  | 1162 | -0.524 | -0.3295 | Yes |
| 191 | RRM1 |  |  | 1166 | -0.534 | -0.3278 | Yes |
| 192 | KIF14 |  |  | 1168 | -0.535 | -0.3243 | Yes |
| 193 | BRCA1 |  |  | 1171 | -0.541 | -0.3217 | Yes |
| 194 | UTRN |  |  | 1178 | -0.551 | -0.3227 | Yes |
| 195 | ORC6 |  |  | 1179 | -0.551 | -0.3181 | Yes |
| 196 | XRCC2 |  |  | 1180 | -0.551 | -0.3135 | Yes |
| 197 | CENPU |  |  | 1182 | -0.555 | -0.3098 | Yes |
| 198 | KIF2C |  |  | 1183 | -0.558 | -0.3052 | Yes |
| 199 | CDCA3 |  |  | 1187 | -0.563 | -0.3033 | Yes |
| 200 | ASPM |  |  | 1189 | -0.567 | -0.2995 | Yes |
| 201 | FEN1 |  |  | 1191 | -0.569 | -0.2957 | Yes |
| 202 | NUF2 |  |  | 1194 | -0.574 | -0.2928 | Yes |
| 203 | DHFR |  |  | 1197 | -0.576 | -0.2898 | Yes |
| 204 | CKS1B |  |  | 1198 | -0.577 | -0.2850 | Yes |
| 205 | GPC6 |  |  | 1204 | -0.589 | -0.2848 | Yes |
| 206 | GTSE1 |  |  | 1210 | -0.602 | -0.2845 | Yes |
| 207 | CLSPN |  |  | 1212 | -0.605 | -0.2804 | Yes |
| 208 | KIF15 |  |  | 1213 | -0.610 | -0.2753 | Yes |
| 209 | CENPK |  |  | 1220 | -0.619 | -0.2757 | Yes |
| 210 | C21orf58 |  |  | 1222 | -0.626 | -0.2714 | Yes |
| 211 | DUT |  |  | 1223 | -0.633 | -0.2662 | Yes |
| 212 | SMC4 |  |  | 1224 | -0.638 | -0.2609 | Yes |
| 213 | MYBL2 |  |  | 1225 | -0.639 | -0.2555 | Yes |
| 214 | CKS2 |  |  | 1226 | -0.641 | -0.2502 | Yes |
| 215 | NCAPH |  |  | 1228 | -0.643 | -0.2458 | Yes |
| 216 | ANLN |  |  | 1229 | -0.646 | -0.2404 | Yes |
| 217 | KIF18A |  |  | 1231 | -0.649 | -0.2359 | Yes |
| 218 | E2F7 |  |  | 1237 | -0.664 | -0.2351 | Yes |
| 219 | CDCA8 |  |  | 1239 | -0.669 | -0.2304 | Yes |
| 220 | CDCA5 |  |  | 1244 | -0.685 | -0.2284 | Yes |
| 221 | AURKB |  |  | 1250 | -0.700 | -0.2273 | Yes |
| 222 | MELK |  |  | 1251 | -0.701 | -0.2214 | Yes |
| 223 | CCNA2 |  |  | 1255 | -0.716 | -0.2183 | Yes |
| 224 | UBE2T |  |  | 1256 | -0.719 | -0.2123 | Yes |
| 225 | CDCA2 |  |  | 1258 | -0.727 | -0.2072 | Yes |
| 226 | ARHGAP11A |  |  | 1262 | -0.735 | -0.2038 | Yes |
| 227 | HMGB2 |  |  | 1263 | -0.736 | -0.1977 | Yes |
| 228 | RAD51AP1 |  |  | 1264 | -0.741 | -0.1915 | Yes |
| 229 | KIF23 |  |  | 1265 | -0.741 | -0.1854 | Yes |
| 230 | KIFC1 |  |  | 1267 | -0.748 | -0.1801 | Yes |
| 231 | TYMS |  |  | 1272 | -0.770 | -0.1774 | Yes |
| 232 | CCNE2 |  |  | 1273 | -0.771 | -0.1710 | Yes |
| 233 | ATAD2 |  |  | 1275 | -0.775 | -0.1654 | Yes |
| 234 | NDC80 |  |  | 1278 | -0.810 | -0.1606 | Yes |
| 235 | SYNE2 |  |  | 1282 | -0.829 | -0.1565 | Yes |
| 236 | ESCO2 |  |  | 1283 | -0.846 | -0.1494 | Yes |
| 237 | FAM111A |  |  | 1285 | -0.871 | -0.1431 | Yes |
| 238 | HJURP |  |  | 1290 | -0.897 | -0.1393 | Yes |
| 239 | SPC25 |  |  | 1294 | -0.932 | -0.1344 | Yes |
| 240 | RRM2 |  |  | 1295 | -0.933 | -0.1266 | Yes |
| 241 | KPNA2 |  |  | 1297 | -0.972 | -0.1194 | Yes |
| 242 | FBXO5 |  |  | 1301 | -0.992 | -0.1140 | Yes |
| 243 | NCAPG |  |  | 1303 | -1.015 | -0.1065 | Yes |
| 244 | HIST1H1E |  |  | 1309 | -1.319 | -0.1001 | Yes |
| 245 | HIST1H1C |  |  | 1310 | -1.391 | -0.0886 | Yes |
| 246 | CDK1 |  |  | 1311 | -1.433 | -0.0766 | Yes |
| 247 | UBE2C |  |  | 1312 | -1.466 | -0.0644 | Yes |
| 248 | TOP2A |  |  | 1313 | -1.528 | -0.0517 | Yes |
| 249 | HIST1H1B |  |  | 1318 | -1.922 | -0.0394 | Yes |
| 250 | HIST1H1D |  |  | 1319 | -1.958 | -0.0231 | Yes |
| 251 | HIST1H4C |  |  | 1320 | -2.769 | -0.0000 | Yes |
Table: GSEA details [plain text format]

  

Fig 2: RICHARDS\_NOWAKOWSKI\_SCIENCE\_2017\_MGE\_IPC2\_UPREG: Random ES distribution      
 Gene set null distribution of ES for **RICHARDS\_NOWAKOWSKI\_SCIENCE\_2017\_MGE\_IPC2\_UPREG**
